# Characterization of Antimicrobial Resistance Determinants and Class 1 and Class 2 Integrons in *Salmonella* *enterica* spp., Multidrug-Resistant Isolates from Pigs

**DOI:** 10.3390/genes9050256

**Published:** 2018-05-16

**Authors:** Héctor Argüello, Beatriz Guerra, Irene Rodríguez, Pedro Rubio, Ana Carvajal

**Affiliations:** 1 Group of Genomics and Animal breeding, Department of Genetics, Faculty of Veterinary Medicine, University of Córdoba, 14071 Córdoba, Spain; 2 Department of Animal Health, Faculty of Veterinary Medicine, University of León, 24007 León, Spain; p.rubio@unileon.es (P.R.); ana.carvajal@unileon.es (A.C.); 3 German Federal Institute for Risk Assessment, 10589 Berlin, Germany; beatriz.guerra@efsa.europa.eu (B.G.); irene.rodriguez.fdez@gmail.com (I.R.); 4 European Food Safety Authority, 43126 Parma, Italy

**Keywords:** resistance gene, swine, food-borne pathogen, public health, antibiotic

## Abstract

Antimicrobial resistance (AMR) and *Salmonella* spp., are primary concerns in public health. The present study characterizes the AMR determinants of 62 multi-drug resistant (MDR) *Salmonella enterica* spp., isolates from swine, which were obtained between 2004–2006, a major source of human salmonellosis. The AMR determinants were investigated by PCR, checking the presence of class 1 and class 2 integrons and 29 resistance genes. Genes *sul1*, *blaTEM1*-like, *aadA2*, *tet*(A), and *dfrA12* were more prevalent (*p* < 0.05) within the determinants that were checked for each of these antimicrobials. Co-existence of different genes conferring resistance to the same antimicrobial was common. No differences in AMR determinants prevalence were observed between *Salmonella* Typhimurium and other serovars from the study. Class 1 integrons were detected in 48 of 62 isolates, again with no differences being linked to any serovar. Nine different variable regions were observed, 1000 bp/*aadA2*-1200 bp/*blaPSE-1* (13 isolates) and bla*OXA*-like/*aadA1* (eight isolates) were the most common. Four isolates, including *S.* Typhimurium (2), *Salmonella* Bredeney (1), and *Salmonella* Kapemba (1) harboured a class 2 integron 2300 bp estX-*sat2-aadA1*. Results from the study highlight the importance of class 1 integrons and certain genes in MDR swine *Salmonella* isolates. The information is of relevance for monitoring in the forthcoming scope of reduction of antibiotic usage in swine production.

## 1. Introduction

The high incidence of human salmonellosis, with an estimated number of 93.8 million illnesses each year [1], highlights the importance of this pathogen among food-borne toxi-infections. Medical attention and even hospitalization could be required in severe cases, children, elderly people, and immunocompromised patients. Antibiotics are frequently used in the treatment of these complicated cases [2] and the emergence of multi-drug resistant (MDR) *Salmonella* spp., isolates is behind the failure of antimicrobial treatments, hindering the recovery of patients and even jeopardizing their life [2,3].

More than 85% of the human salmonellosis cases are transmitted by contaminated foodstuffs [1] with chicken, eggs, and pork among the most common sources of infection [4]. The success of avian *Salmonella* control programmes has positively impacted in the incidence of the infection in the EU [5]. The reduction of cases linked to chicken or eggs consumption has indirectly increased the relative relevance of pork products, since not all countries are implementing control measures in swine production [6] or measures that are taken in some others are not achieving the expected results [7]. Moreover, multi-drug resistance in *Salmonella* spp., isolated from swine is a quite common feature [8,9,10]. Firstly, the use of antibiotics as growth promoters and after the ban of this practice (Regulation EC 1831/2003), their use in prophylactic and metaphylactic treatments has positively enhanced the selection of MDR strains, particularly in *Salmonella* Typhimurium isolates [11], the main *Salmonella* serotype that is associated to swine production [12,13]. The huge concern about these “super-bugs” has led to new recommendations with the aim of reducing and replacing the use of antimicrobials in animal production [14,15].

Next-future animal farming will for sure change the production systems to reduce the use of antimicrobials. Theoretically, limiting or even removing the use of antibiotics will decrease the presence of antimicrobial resistance (AMR) in microorganisms, such as *Salmonella*, either by losing the AMR determinants that are located in mobile elements such as plasmids, or by the replacement of the strains that are circulating on farms by others that adapt better to a new antibiotic-free scenario. The large number of studies that are focused on phenotypic characterization of AMR in *Salmonella* isolates recovered from swine [9,16,17,18], contrast to the scarce information about the genetic determinants that confer resistance to these antimicrobial compounds [19,20]. While considering the forthcoming scenario, more information about the genetic determinants in *Salmonella* is required to better understanding the mechanisms which confer these AMR, their potential spread and how an antibiotic-free animal production system would impact in the reduction of MDR bacteria in farm animals.

To achieve this goal, the present study characterizes the AMR determinants as well as the presence of class 1 and class 2 integrons in a collection of 62 MDR *Salmonella* isolates that were obtained in two cross-sectional studies [21,22] carried out in finishing pigs in Spain.

## 2. Materials and Methods

### 2.1. Bacterial Isolates

Sixty-two multi-resistant *Salmonella* isolates were included in this study (Table 1). The collection of MDR isolates comes from (1) a cross-sectional study in finishing pigs, collecting faecal pools from pen floors (2004) [22], and (2) another cross-sectional study to estimate the within country prevalence in member states of the European Union, in which mesenteric lymph nodes were the target sample (2006–2007) [21]. Serotyping of *Salmonella* isolates was performed by slide-agglutination, as described elsewhere [23], following the Kauffman-White-Le Minor scheme [24].

All 62 isolates were resistant to a minimum of three antimicrobial compounds and they belonged to nine different serotypes: *S.* Typhimurium (43 isolates), the monophasic variant of *S.* Typhimurium *S.* 4,[5],12: i:- (5 isolates), *Salmonella* Derby (5 isolates), *Salmonella* Rissen (3 isolates), *Salmonella* Bredeney (2 isolates), *Salmonella* London (1 isolate), *Salmonella* Kapemba (1 isolate), *Salmonella* Wien (1 isolate), and *Salmonella* Worthington (1 isolate). Isolates were obtained from different farms or abattoirs that were spread all over the country and had no apparent epidemiological link.

### 2.2. Phenotypic Characterization of Antimicrobial Resistance Genes

Each isolate was tested with a panel of 17 antimicrobials using custom-defined microtiter plates (TREK Diagnostic Systems IncEast Grinstead, West Sussex, UK), including amoxicillin (AMC), ampicillin (AMP). apramycin (APR), cephalothin (CEP), ceftiofur (XNL), ciprofloxacin (CIP), chloramphenicol (CHL), colistin (COL), florphenicol (FFN), gentamicin (GEN), nalidixic acid (NAL), neomycin (NEO), spectinomycin (SPE), streptomycin (STR), sulphamethoxazole (SMX), trimethoprim/sulfamethoxazole (TMP), and tetracycline (TET). Panels were read after 24 h incubation and minimal inhibitory concentration (MIC), which was defined as the lowest concentration that inhibited visible bacterial growth, was determined. Isolates were classified as resistant using the Clinical and Laboratory Standards Institute (CLSI) breakpoints [25].

### 2.3. Detection of Antimicrobial Resistance Determinants

Detection of the AMR determinants was carried out by PCR of target genes. The following genes encoding resistance to ampicillin: *blaPSE-1*, *blaTEM1*-like and *blaOXA1*-like; chloramphenicol: *catA1*, *cmlA* and *floR*; gentamicin: *aac(3)-IIa*, *aac(3)-IVa and aadB*; kanamycin: *aphA1* and *aphA2*; fluoroquinolones: *qnrS*, *qnrB*, *qnrC*, *qnrD*; streptomycin: *aadA1*-like and *aadA2*, sulfamethoxazole: *sul1*, *sul2* and *sul3*; *tet*racycline: *tet*(A), *tet*(B), and *tet*(G); and, trimethoprim: *dfrA1*-like, *dfrA5*-A*14*, *dfrA*-*17*, *dfrA12*, and *dfrA7-A17* were screened as previously described [11]. PCR primers, conditions and products size are detailed in Table S1.

### 2.4. Identification of Class 1 and Class 2 Integrons

The presence of class 1 and class 2 integrons was investigated by the amplification of the variable region (VR) using the 5’-CS/3’-CS primers that anneal with sequences flanking the *att1* site of class 1 integrons and Hep74/Hep51 primers that bind to *att2* and ORFX within class 2 integrons [26]. A detailed description of PCR procedures is shown in Table S1.

### 2.5. Statistical Analysis

Data were introduced into R (R-project) [27] for further statistical analysis. Differences in prevalence of genetic determinants were assessed using a Χ^2^ or Fisher’s exact test. Significance of statistical analysis was established at α = 0.05. 

## 3. Results

### 3.1. Antimicrobial Resistance Profiles within the Multi-Drug Resistance isolates

The 62 isolates that were included in this study exhibited 47 MDR profiles. The number of antimicrobial resistances per isolate varied from three up to 14 (Table 1). Most of the MDR profiles (39) were associated to a single isolate, while only two of them were shared by four or more isolates: the tetra-resistant profile AMP-SPE-STR-TET detected in *S.* Typhimurium (four isolates) and the hepta-resistant profile AMP-CHL-FFN-SMX-SPE-STR-TET found in *S.* Typhimurium (six isolates). Only one MDR profile (AMP-APR-CEP-CHL-GEN-SMX-SPE-STR-TET-TMP) was found in isolates from two different serotypes, *S.* Typhimurium (1) and *Salmonella* 4,[5],12:i:- (1).

### 3.2. Frequency of the Antimicrobial Resistant Determinants

The characterization of the determinants that were responsible for the MDR profiles included the identification of 29 genes that were responsible for resistance to nine antibiotics (Table 2, Supplementary Table S2). All of the isolates that were resistant to nalidixic acid, 13 in total, were negative to the *qnr* genes checked. A limited number of isolates were resistant to the aminoglycosides kanamycin and gentamicin. The four resistant isolates to kanamycin were associated to the presence of the gene *aphAI*. The gene *aac3-IVa* was detected in 16 of the 17 resistant isolates to gentamicin (94.1%), *aac3-IIa* in the other isolate (5.9%), while none of the gentamicin resistant isolates were positive to the gene *aadB*. In contrast, resistance to the two other aminoglycosides tested, spectinomycin and streptomycin, was the most frequent resistance observed among the antimicrobials that were included in this study, with 53 of the 62 isolates resistant to spectinomycin and streptomycin. The genes *aadA1*-like and *aadA2* were identified in 48 (90.6%) and 35 (66%) of these resistant isolates, respectively, a difference that was statistically significant (*p* = 0.015).

**Figure 1:**
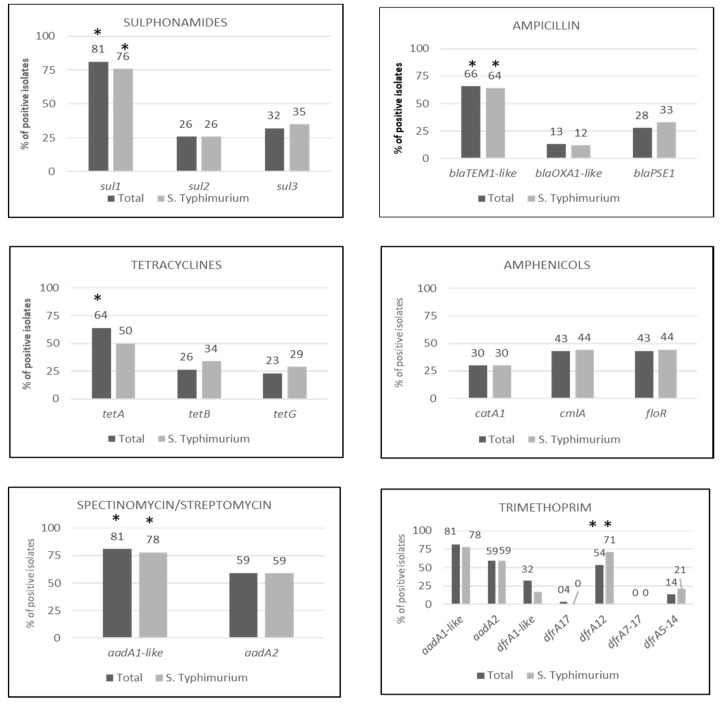
Percentage (%) of positive isolates to each resistance gene for SMX, AMP, TET, CHL, SPE/STR and TMP in a collection of 62 multi-drug resistant *Salmonella* isolates that were recovered from swine. Black bars include the 62 isolates, while grey bars report the same information only for the 43 *S.* Typhimurium isolates. Significant differences (*p* < 0.05) in prevalence of a certain genetic determinant within the collection or for S. Typhimurium isolates is denoted with an asterisk above the back or grey bars, respectively (*).

Resistance to sulphonamides and tetracyclines (53 isolates each) and penicillins (47 isolates) was also common within the collection of isolates. We observed significant dissimilarities in the frequency of the genes that were responsible for the AMR to these antimicrobials, as well as for the resistance to trimethoprim (Figure 1).

Sulphametoxazole resistant isolates harbored *sul1*, *sul2*, *sul3*, or a combination of the three genes (19 isolates); *sul1* was present in 43 isolates (81.1% of the isolates resistant to sulphametoxazole), all of them as part of class 1 integrons. The frequency of *sul1* was significantly higher (*p* < 0.001) than the other two sulphametoxazole resistance determinants that were investigated (Figure 1). Interestingly, a significant number of isolates (18) carried two different genes conferring resistance to sulphametoxazole.

The gene *tet*(A) was reported in 34 isolates (64.15% of the isolates resistant to tetracycline), and was more prevalent than the other two genes conferring resistance to this antimicrobial, *tet*(B), and *tet*(G) (*p* < 0.001). The gene *tet*(G) was found in 12 isolates, nine of which presented the penta-resistance profile characteristic of the *Salmonella* genomic island 1 (SGI-1).

Among genes that were conferring resistance to penicillins (ampicillin), *blaTEM1*-like was detected in 34 isolates (65.96% of ampicillin resistant isolates) being more frequent than the other two ampicillin resistance determinants tested, *blaOXA1*-like, and *blaPSE-1* (*p* < 0.01).

When only the *S.* Typhimuriun isolates were evaluated, *sul1* (*p* < 0.001), *blaTEM1*-like (*p* < 0.001), *tet*(A) (*p* < 0.001), and *dfrA12* (*p* < 0.001) were also more prevalent within their respective antimicrobial groups (Figure 1). The frequency of resistance determinants in the *S*. Typhimurium group was also compared with the rest of the isolates. The gen *tet*(A) was more prevalent in other serotypes when compared to *S.* Typhimurium (*p* < 0.001). 

### 3.3. Integrons

Class 1 integrons were detected in 48 of the 62 MDR *Salmonella* isolates (77%). Table 3 summarizes the nine different VRs for the class 1 integrons detected, including defective integrons. The VR, including the cassette 1000 bp/*aadA2*-1200 bp/*blaPSE-1*, was the most common (13 isolates) while the 2 kb VR *blaOXA*-like/*aadA1*, detected in eight isolates, was the second most frequent VR. Another six isolates carried the 1 kb VR harboring *aadA1*-like cassette. Three different VRs of 1.6 kb were also detected, *dfrA1*/*aadA1* cassettes (three isolates), *dfrA17*/*aadA5* (1 isolate) and *aac3-IV*/*aadA7* (1 isolate). In addition to the cassettes that were detected in the VR or within the integron, all these isolates presented several AMR genes, some of which were present in all isolates carrying the same class 1 integron (Table 3).

Class 1 integrons were detected in 31 of 43 *S.* Typhimurium isolates and 17 of 19 non-Typhimurium isolates. These differences in the frequency of class 1 integrons did not reach statistical significance (*p* = 0.11). From the VRs that were detected, it is noteworthy that the combination of VRs 1000 bp/*aadA2*, together with 1200 bp/*blaPSE-1*, was limited to *S.* Typhimurium, along with the presence of 200 bp VR and defective integrons.

Class 2 integrons were only detected in four isolates, all with the same VR size, 2300 bp/estX-*sat2-aadA1* (Table 3). These class 2 integrons were found in three different serotypes, *S.* Typhimurum (2), *S.* Bredeney (1), and *S.* Kapemba (1).

## 4. Discussion

Increased resistance to antimicrobials in foodborne pathogens is a major concern for authorities, which have focused their efforts on limiting the use of antibiotics in animal production [28]. According to the European Centre for Disease Prevention and Control (ECDC), European Food Safety Authority (EFSA), and European Medicines Agency (EMA) scientific opinion [28], *Salmonella* has been considered as a priority microorganism for monitoring AMR. Characterizing the genes that are responsible for this resistance and their spread is necessary to evaluate the impact of the strategies that are put in place to reduce the use of antibiotics in the persistence of AMR determinants in *Salmonella* spp. Despite the lapse of time between the collection of the isolates and the study, the characterization of isolates from cross-sectional studies is a great opportunity to establish a reference for future studies.

Acquisition and spreading of genetic determinants of AMR in *Salmonella* mainly occurs by the horizontal transference of mobile elements, such as plasmids [29]. As mostly being carried by plasmids or contained within transposons, integrons are genetic elements involved in the spread of resistance by the inclusion of AMR gene cassettes [30,31]. Integrons are divided into classes based on the amino acid sequence of the integrase gene (*intI*) [31]. Class 1 integrons are the most commonly integron reported among *Salmonella* clinical isolates [26,32], with a variable prevalence among countries and sources [30]. In this study, almost four of each five multi-resistant *Salmonella* isolates harbored class 1 integrons. Previous studies with *Salmonella* isolates from swine that were carried out in Vietnam (24.5%), or more recently in China (28%), reported lower ratios of class 1 integrons [20,30]. Similar studies in *Salmonella* spp., which was isolated from Spanish poultry, reported no incidence of class 1 integrons within the isolates collection [33]. It could be argued that the high prevalence in class 1 integrons detected is consequence of the pre-selection of MDR isolates to perform our study, but even with this premise, the prevalence is notably higher than was reported in other studies with a similar approach [34,35]. Swine production is foremost in antimicrobial consumption across farm animals [4]. That is a potential selection pressure for the strains that are characterized in our study. These results are of interest for future studies monitoring the prevalence of this integron class in *Salmonella* isolates from swine.

Up to nine different VRs for class 1 integrons were detected. The VR containing the 1000 bp/*aadA2* and 1200 bp/*blaPSE-1* cassettes was the most frequently observed. This result goes in line with a previous study that was carried out with clinical and foodstuff *Salmonella* isolates that were obtained in the 90s [36]. The other two most common VRs observed, 1000 bp/*aadA1* and 2,000 bp/*blaOXA1*-like-*aadA1*, were also reported in previous studies, including clinical isolates in Spain [26,35]. These results evidence that similar VRs are circulating among human and food animal *Salmonella* strains despite the lapse of time between these studies. The variability of cassette arrays that were detected in this study, together with their presence in different serotypes, is a proof of the contribution of class 1 integrons in the dissemination of AMR gene cassettes in different *Salmonella* serotypes, as previously reported [11,35,37]. Moreover, in our study, eight isolates carried defective integrons, lacking part of the classical class 1 integron structure [11]. Defective integrons may be disregarded if no particular seach is performed. The frequency that was observed in our study suggest the particular search of them this sort of studies.

Class 2 integrons structure is similar to class 1 integrons, and they are also involved in the dissemination of AMR [30]. However, class 2 integron information is limited compared to class 1 integrons [26], and their presence in *Salmonella* isolates has only been reported by a few studies [26,34,38,39]. A class 2 integron with a VR of approximately 2300 bp (*estX*-*sat2*-*aadA1*) was identified in *S.* Typhimurium, *S.* Bredeney, and *S.* Kapemba isolates. The presence of class 2 integrons has been reported in serotypes of relevance from a public health perspective such as *S.* Typhimurium [40], as well as in other non-prevalent *Salmonella* serovars, such as *S.* Bredeney, *Salmonella* Panama, *Salmonella* Virchow, or *Salmonella* Heidelberg [26,34,38]. In our study, a new non-prevalent serotype has been added to the list, reflecting that exotic serovars, such as *S.* Kapemba, can also carry class 2 integrons in the production animal species, such as swine.

Antimicrobial resistance patterns varied from three to 14 resistances. The fact that most of the resistance profiles were associated to a single isolate evidence the wide heterogenicity of the strains used in the study. Although serovars of importance in swine are well represented within the collection, current monitoring studies have noticed an increase of serovars, such as the monophasic variant of *S.* Typhimurium (S. 4,[5],12:i:-) [10], which may denote differences in AMR determinants in future studies. The genes *sul1*, *aad1*-like, *blaTEM1*-like, *tet*(A), and *dfrA-12* were more frequent among the MDR isolates. Except for *tet*(A), the same differences were observed when only the *S.* Typhimurium isolates were included in the analysis. Among them, *sul1* and *aad1*-like determinants were associated to integrons. TEM-like genes, such as *blaTEM1*-like, are the most frequent conferring resistance to ampillicin in *Salmonella* [34]. Regarding tetracycline resistance, *tet*(B) is considered to be the most common determinant in *Salmonella* [41]. The higher proportion of *tet*(A) that was reported in our study can be associated particularly to *Salmonella* isolates from Spanish swine. Previous studies have shown the ability of *Salmonella* to transfer class 1 and class 2 integrons as well as AMR determinants [30,34]. The fact that no differences in AMR determinants were detected between *S.* Typhimurium, a recognized MDR serotype, and the other serotypes included in the study supports the horizontal transference of the determinants, regardless of their serotype.

Chloramphenicol is no longer used in veterinary medicine [22], but the frequency of resistance determinants against chloramphenicol is still high, associated to the vertical transmission in genetic elements, such as the SGI-1. The penta-resistance pattern of the SGI-1 *blaPSE-1*, *floR*, *aadA2*, *sul1*, and *tet*(G) [11] was found in nine *S.* Typhimurium isolates, which plausibly harbour this genomic island. If *Salmonella* is going to be included in monitoring studies for the evaluation of antimicrobials reduction programs, attention should be paid to the mechanisms of transference of the target genes, as they will influence the final outcome and conclusions. In line with this last idea, another feature quite common within the MDR isolates included in our study, was the co-existence of resistance determinants for the same antimicrobial. Two genes conferring resistance to sulphonamides and spectinomycin-streptomycin were present in a large number of strains. Co-existence of *aadA* genes has already been reported, but the presence of different *sul* genes within the same isolate seems to be uncommon, above all, if the *sul3* determinant is involved [42]. Co-presence of several determinants conferring resistance to the same antimicrobial might slow down the removal of MDR strains in an antibiotic-free or antibiotic-limited animal farming as may be needed more than one genetic event to lose those genes.

No plasmid-mediated quinolone resistance was detected in any of the MDR strains that were included. Fluoroquinolones are the antimicrobial of choice in human salmonellosis [22] and the presence of horizontal transmission determinants that confer resistance to this group of antimicrobial drugs vary among studies [37,43].

These results are the first genotypic characterization of AMR genes in *Salmonella* isolates from Spanish swine. Their value are not only to understand the mechanisms of resistance, but also for future studies monitoring the evolution of resistance gene in *Salmonella* from production animals, of course carefulness about that only MDR strains are included in our study. The implementation of whole genome sequencing in the AMR monitoring will increase the frequence of reports in AMR determinats, which are necessary to understand the evolution of the mechanisms that are used by the bacteria to overcome the antibiotics [44].

In conclusion, the analysis of genetic determinants in a collection of MDR *Salmonella* isolates revealed the involvement of class 1 integrons in the spread and acquisition of different gene cassettes, not only in *S.* Typhimurium, but also in other serotypes that are circulating on swine farms. High frequency of certain AMR determinants demonstrates their transmission capacity. The fact that no differences were observed in their frequencies between *S.* Typhimurium and other serovars also points towards the relevance of all *Salmonella* serovars circulating on swine farms. The results of this study, genetic determinants and integrons present as well as their frequency, are of interest in future research on the effect of antimicrobial reduction or the removal in AMR carriage in *Salmonella* from swine production.

## Figures and Tables

**Table 1:** Antimicrobial resistance patterns detected in a collection of 62 multi-drug resistant *Salmonella* isolates that were recovered from swine.

No. of Isolates	Antimicrobial Resistance Pattern	Serotype(s)
1	AMP-SMX-TMP	*S.* 4,[5],12:i:-
1	SPE-SMX-TET	*Salmonella* Typhimurium
1	AMP-SMX-SPE-STR	*S.* Typhimurium
4	AMP-SPE-STR-TET	*S.* Typhimurium
2	SMX-SPE-STR-TET	*S.* Typhimurium
1	AMP-SMX-SPE-STR-TMP	*S.* Typhimurium
1	AMP-SPE-STR-TET-TMP	*S.* Typhimurium
1	CHL-SMX-SPE-STR-TET	*S.* Typhimurium
2	FOT-SMX-SPE-STR-TET	*Salmonella* Derby
1	SMX-SPE-STR-TET-TMP	*S.* Typhimurium
1	AMP-CHL-FFN-SPE-STR-TET	*S.* Typhimurium
1	AMP-CHL-NEO-SPE-STR-TET	*S.* Typhimurium
2	AMP-CHL-SMX-SPE-STR-TET	*S.* Typhimurium
1	AMP-SMX-SPE-STR-TET-TMP	*Salmonella* Bredeney
1	CHL-CIP-FFN-GEN-NAL-SMX	*S.* Typhimurium
6	AMP-CHL-FFN-SMX-SPE-STR-TET	*S.* Typhimurium
3	CHL-FFN-SMX-SPE-STR-TET-TMP	*S.* Typhimurium
1	AMP-CIP-FOT-NAL-SMX-SPE-STR	*S.* Typhimurium
1	AMP-FOT-KAN-GEN-SPE-STR-TET	*S.* Typhimurium
1	AMP-FOT-NEO-SMX-SPE-STR-TET	*S.* Derby
1	AMP-FOT-NEO-SMX-SPE-STR-TMP	*S.* Derby
1	AMP-FOT-SMX-SPE-STR-TET-TMP	*Salmonella* Rissen
1	APR-CHL-FFN-GEN-SMX-SPE-STR-TET	*S.* Derby
1	AMP-APR-CEP-GEN-SMX-SPE-STR-TET	*S.* Typhimurium
1	AMP-CEP-CHL-SMX-SPE-STR-TET-TMP	*S.* Rissen
2	AMP-CHL-CIP-NAL-SMX-SPE-STR-TET	*S.* Typhimurium
1	AMP-CHL-FFN-NAL-SMX-SPE-STR-TET	*S.* Typhimurium
1	AMP-CHL-FFN-SMX-SPE-STR-TET-TMP	*S.* Typhimurium
1	AMP-CHL-NAL-SMX-SPE-STR-TET-TMP	*Salmonella* Kapemba
1	AMP-CEP-CIP-SMX-SPE-STR-TET-TMP	*Salmonella* Worthington
1	AMP-GEN-KAN-SMX-SPE-STR-TET-TMP	*S.* Typhimurium
1	CHL-CIP-FFN-GEN-NAL-SMX-SPE-STR	*S.* Typhimurium
1	CHL-CIP-NAL-SMX-SPE-STR-TET-TMP	*Salmonella* 4,[5],12:i:-
1	AMP-APR-CEP-GEN-SMX-SPE-STR-TET-TMP	*S.* 4,[5],12:i:-
1	AMP-CEP-COL-GEN-SMX-SPE-STR-TET-TMP	*S.* Typhimurium
1	AMP-CHL-FOT-GEN-KAN-SMX-SPE-STR-TET	*Salmonella* London
1	AMP-CHL-CIP-FFN-FOT-NAL-SMX-SPE-STR-TET	*S.* Typhimurium
1	AMP-CHL-GEN-KAN-SMX-SPE-STR-TET-TMP	*S.* Rissen
1	CHL-CIP-FFN-NAL-SMX-SPE-STR-TET-TMP	*S.* Bredeney
1	AMP-CHL-CIP-FFN-NEO-SMX-SPE-STR-TET-TMP	*S.* Typhimurium
2	AMP-APR-CEP-CHL-GEN-SMX-SPE-STR-TET-TMP	*S.* Typhimurium/*S.* 4,[5],12:i:-
1	AMP-APR-CHL-FFN-GEN-SMX-SPE-STR-TET-TMP	*S.* Typhimurium
1	AMP-CEP-CHL-CIP-NAL-NEO-SMX-SPE-STR-TMP	*Salmonella* Wien
1	AMP-CHL-CIP-FFN-FOT-NAL-NEO-SPE-STR-TET	*S.* Typhimurium
1	AMP-CHL-CEP-GEN-SPE-SMX-SPE-STR-TET-TMP	*S.* Typhimurium
1	AMP-APR-CEP-CIP-CHL-FFN-GEN-NAL-SMX-SPE-STR-TET-TMP	*S.* 4,[5],12:i:-
1	AMP-APR-CEP-CHL-CIP-FFN-GEN-NAL-NEO-SMX-SPE-STR-TET-TMP	*S.* Typhimurium

AMC: amoxicillin; AMP: ampicillin; APR: apramycin; CEP: cephalothin; XNL: ceftiofur; CIP: ciprofloxacin; CHL: chloramphenicol; COL: colistin; FFN: florphenicol; GEN: gentamicin; NAL: nalidixic acid; NEO: neomycin; SPE: spectinomycin; STR: streptomycin; SMX: sulphamethoxazole; TMP: trimethoprim/sulfamethoxazole; TET: tetracycline.

**Table 2:** Frequencies of antimicrobial resistance determinants examined in a collection of 62 multi-drug resistant *Salmonella* isolates recovered from swine.

Antimicrobial Compound ^1^ (Genes Tested)	*S.* Typhimurium	*S.* 4,[5],12:i:-	*S.* Derby	*S.* Rissen	*S.* Bredeney	*S.* Kapemba	*S.* London	*S.* Worthington	*S.* Wien
Sulphonamides	34 isolates	5 isolates	5 isolates	3 isolates	2 isolates	1 isolate	1 isolate	1 isolate	1 isolate
*sul1*	26 (76.5%)	5 (100%)	4 (80%)	3 (100%)	1 (50%)	1 (100%)	1 (100%)	1 (100%)	1 (100%)
*sul2*	9 (26.5%)	2 (40%)	1 (20%)	1 (33%)	1 (50%)	0 (0%)	0 (0%)	0 (0%)	0 (0%)
*sul3*	12 (35.2%)	2 (40%)	0 (0%)	1 (33%)	1 (50%)	0 (0%)	0 (0%)	0 (0%)	1 (100%)
Ampicillin	33 isolates	4 isolates	2 isolates	3 isolates	1 isolate	1 isolate	1 isolate	1 isolate	1 isolate
bla*TEM1*-like	21 (63.6%)	3 (75%)	1 (50%)	2 (66%)	1 (100%)	1 (100%)	1 (100%)	0 (0%)	1 (100%)
bla*OXA1*-like	4 (12.1%)	0 (0%)	1 (50%)	1 (33%)	0 (0%)	0 (0%)	0 (0%)	0 (0%)	0 (0%)
bla*PSE-1*	11 (33.3%)	1 (25%)	0 (0%)	1 (33%)	0 (0%)	0 (0%)	0 (0%)	0 (0%)	0 (0%)
Tetracycline	38 isolates	4 isolates	4 isolates	3 isolates	1 isolate	1 isolate	1 isolate	1 isolate	0 isolates
*tet*(A)	19 (50%)	4 (100%)	4 (100%)	3 (100%)	1 (100%)	1 (100%)	1 (100%)	1 (100%)	-
*tet*(B)	13 (34.2%)	0 (0%)	1 (25%)	0 (0%)	0 (0%)	0 (0%)	0 (0%)	0 (0%)	-
*tet*(G)	11 (28.9%)	0 (0%)	0 (0%)	0 (0%)	0 (0%)	0 (0%)	1 (100%)	0 (0%)	-
Amphenicols	27 isolates	3 isolates	1 isolate	2 isolates	1 isolate	1 isolate	1 isolate	0 isolates	1 isolate
*catA1*	8 (29.6%)	1 (33.3%)	0 (0%)	0 (0%)	0 (0%)	1 (100%)	1 (100%)	-	0 (0%)
*cmlA*	12 (44.4%)	2 (66.6%)	0 (0%)	1 (50%)	0 (0%)	0 (0%)	0 (0%)	-	1 (100%)
*floR*	12 (44.4%)	1 (33.3%)	1 (100%)	1 (50%)	1 (100%)	0 (0%)	0 (0%)	-	0 (0%)
Spectinomycin/Streptomycin	41 isolates	4 isolates	5 isolates	3 isolates	2 isolates	1 isolate	1 isolate	1 isolate	1 isolate
*aadA1*-like	32 (78%)	3 (75%)	4 (80%)	3 (100%)	2 (100%)	1 (100%)	1 (100%)	1 (100%)	1 (100%)
*aadA2*	24 (58.5%)	4 (100%)	4 (80%)	0 (0%)	0 (0%)	1 (100%)	0 (0%)	0 (0%)	0 (0%)
Trimethoprim	14 isolates	5 isolates	1 isolate	3 isolates	2 isolates	1 isolate	0 isolates	1 isolate	1 isolate
*dfrA1*-like	2 (16.7%)	1 (20%)	1 (100%)	1 (33%)	2 (100%)	1 (100%)	-	0 (0%)	1 (100%)
*dfrA17*	0 (0%)		0 (0%)	0 (0%)	0 (0%)	0 (0%)	-	1 (100%)	0 (0%)
*dfrA12*	10 (71.4%)		0 (0%)	1 (33%)	0 (0%)	0 (0%)	-	0 (0%)	0 (0%)
*dfrA7-A17*	0 (0%)		0 (0%)	0 (0%)	0 (0%)	0 (0%)	-	0 (0%)	0 (0%)
*dfrA5-A14*	3 (21.4%)		0 (0%)	0 (0%)	0 (0%)	0 (0%)	-	0 (0%)	0 (0%)
Kanamycin	2	0	0	1	0	0	1	0	0
*aphAI*	2 (100%)		-	1 (100%)	-	-	1 (100%)	-	-
*aphA2*	0 (0%)		-	0 (0%)	-	-	0 (0%)	-	-
Gentamicin	10	3	1	1	0	0	1	0	0
*acc(3)-Iva*	10 (100%)	3 (100%)	1 (100%)	0 (0%)	-	-	1 (100%)	-	-
*acc(3)-Iia*	0 (0%)	0 (0%)	0 (0%)	1 (100%)	-	-	0 (0%)	-	-
*aadB*	0 (0%)	0 (0%)	0 (0%)	0 (0%)	-	-	0 (0%)	-	-

**^1^** Quinolones are not included in the table as all the isolates resistant to NAL were negative to the quinolone resistance genes tested (*qnrS*, *qnrB*, *qnrC*, *qnrD*).

**Table 3:** Class 1 and class 2 integrons detected in a collection of 62 multi-drug resistant *Salmonella* isolates recovered from swine.

Amplicon Size/Resistance Gene	Gene Cassettes Detected in the Isolates Carrying the Class 1 and Class 2 Integrons ^a^	No of Isolates	Serotypes
**Class 1 integrons**			
1000-bp/*aadA1*	*qac sul1 aadA1*-like	6	*S.* Typhimurium (2), *S.* Derby (2), *S.* Kapemba (1) *S.* London (1)
1000-bp/*aadA2*	*qac sul1 aadA2*	3	*S.* Typhimurium (2), *S.* Derby (1)
1000-bp/*aadA2*;1,200-bp/bla*PSE-1*	*qac sul1 blaPSE-1 aadA1*-like *aadA2*	13	*S.* Typhimurium (11), *S.* Rissen (1) *S.* 4,[5],12:i:- (1)
1600-bp/*dfrA1-aadA1a*	*qac sul1 aadA1-like dfrA1*-like	3	*S.* Wien (1), *S.* Rissen (1) and *S.* Bredeney (1)
1600-bp/dfrA17-aadA5	*qac sul1 aadA1*-like	1	*S.* Worthington
1600-bp/aac(3)-aadA7	*qac sul1 aadA1-like dfrA1*-like *aac3*	1	*S.* Typhimurium (1)
1900 bp/ dfrA12-orfF-aadA2	*qac sul1 aadA2 dfrA12*	3	*S.* Typhimurium (2), *S.* 4,[5],12:i:- (1)
2000-bp/bla*OXA*-*aadA1* ^c^	*qac sul1 blaOXA-like aadA1*-like *dfrA12*	6	*S.* Typhimurium (4), *S.* Derby (1), *S.* Rissen (1)
200-bp	*qac sul1*	4	*S.* Typhimurium (1) *S.* 4,[5],12:i:- (3)
Defective^a^,^b^	*aadA1*-like *aadA2 dfrA12*	8	*S.* Typhimurium (8)
**Class 2 integrons**			
2300 bp/ *estX-sat2-aadA1*	*qac sul1 aadA1*-like	4	*S.* Typhimurium (2), *S.* Kapemba (1), *S.* Bredeney (1)

**^a^** Defective integron, undetectable by 5’ CS/3’ CS primers. PCR products were observed using the following primer sets: *int1-R* + *dfrA12*-R; **^b^** Presence of empty variable regions class1 integrons as well.

## Supplementary Materials

The following are available online at http://www.mdpi.com/2073-4425/9/5/256/s1, Table S1: Primers used to amplify the target genes in this study. Table S2: 62 *Salmonella* spp-. strains included in the study, the antimicrobial resistance profile and genetic determinants conferring the resistance.
